# Editorial: From farm gate to food plate: current challenges in foodborne microorganism detection, epidemiology, and genetic diversity

**DOI:** 10.3389/fgene.2023.1320057

**Published:** 2023-10-18

**Authors:** Vinicius Silva Castro, Eduardo Eustáquio de Souza Figueiredo

**Affiliations:** ^1^ Faculty of Agronomy and Zootechnics, Federal University of Mato Grosso (UFMT), Cuiabá, Brazil; ^2^ Faculty of Nutrition, Federal University of Mato Grosso (UFMT), Cuiabá, Brazil

**Keywords:** OneHealth, *campylobacter*, shiga toxin-producing *Escherichia coli*, *brucella*, *pseudomonas*

In recent years, researchers have gained a better understanding of the need to control the entire food production chain to ensure that food products are not contaminated (Lamas et al., 2019; Castro et al., 2022). Several studies have addressed methods and research that span from the place of production to the final consumer’s plate (Julien-Javaux et al., 2019; Nicastro and Carillo, 2021). It should also be noted that researchers have recently emphasized the need for a Onehealth vision, involving the integration of living beings and the way food is produced, in various aspects (Garcia et al., 2020).

**FIGURE 1:**
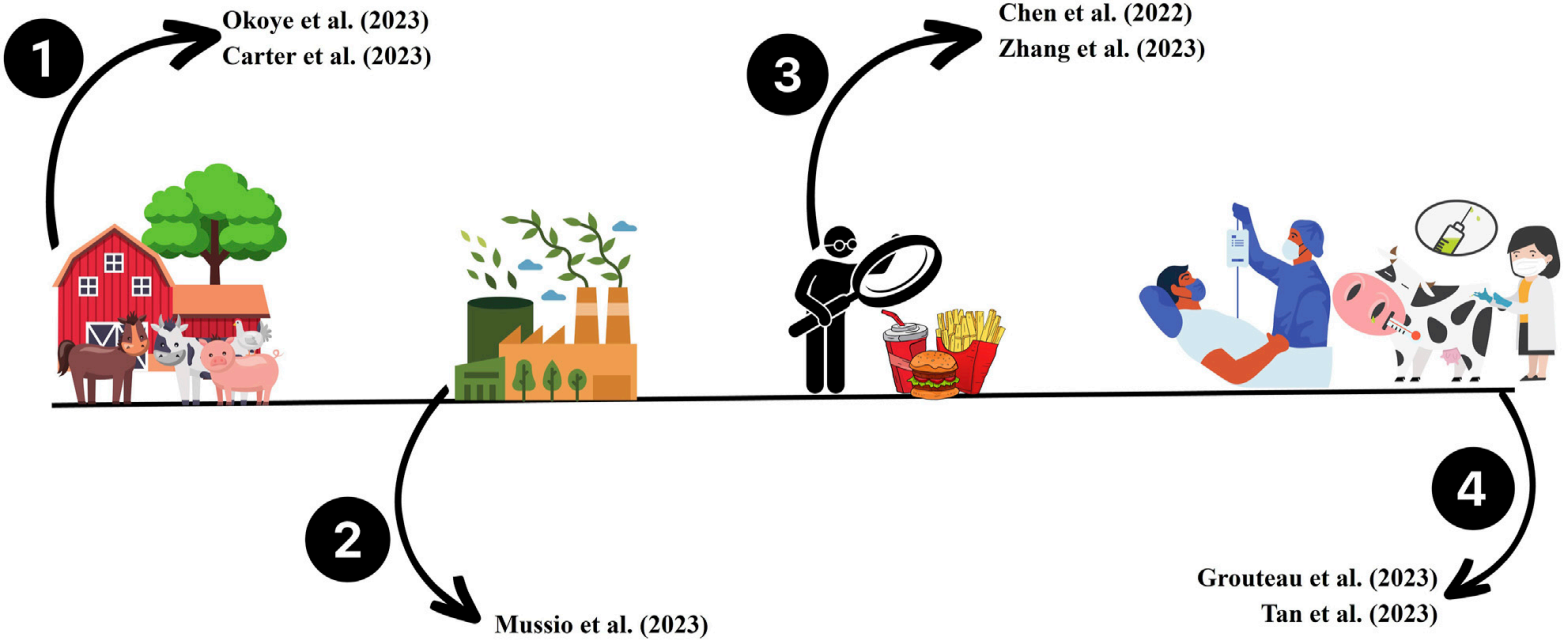
Categorization of articles published in the Research Topic in four established points. The numbers represent the four points of the food chain that we aim to cover: 1 farm or production field; 2 processing and processing industry; 3 Contamination control and monitoring methods; and 4 food plate and consumers affected by foodborne pathogens, or animal diseases.

In this Research Topic proposed by researchers Dr. Eduardo Figueiredo, Dr. Ricardo Carvalho, and Dr. Vinicius Castro, we aim to expand the focus from a traditional Research Topic to a global perspective that encompasses all stages of production. To achieve this, we have divided our attention into four major points, as depicted in Figure 1.

Starting with point 1, the farm, we highlight the study performed by Okoye et al., in which the authors demonstrated that the use of Ferric quinate (QPLEX) has the potential to competitively inhibit contamination with *Campylobacter jejuni* in the interaction of major outer membrane protein (MOMP) with the Lewis b (Leb) antigen. Thus, this study highlights that the results found may be a future alternative to the preventative use of antibiotics in broiler farming to combat *C. jejuni* infection.

Furthermore, another study aimed at identifying potential pathogens in food production was conducted by Carter et al.. In this study, the authors investigated the presence of Shiga toxin-producing *Escherichia coli* (STEC) strains isolated from wild birds in an agricultural region in California. The findings of this study highlighted the possibility of the dissemination of strains with high cytotoxic capacity and strong biofilm formation, suggesting a potential vector of contamination. The presence of these isolates raises concerns in production about the risks of cross-contamination between different species within an agricultural production system.

Regarding point 2, the industry, our Research Topic features an article in which Mussio et al. conducted the first characterization of STEC strains in Uruguay. This study is of utmost importance, considering that Uruguay shares borders with Brazil (one of the largest beef exporters in the world) and Argentina (a country with the most cases of HUS derived from STEC). The results from this study revealed a variety of serogroups non-O157, resulting the most frequent serotypes: O130:H11 (six strains), O174:H28 (5), and O22:H8 (5), present in Uruguay, along with virulence genes of interest in the field of health. We hope that this study will contribute to understanding the dynamics of STEC contamination in the Latin American region.

Regarding point 3, on detection methods, this Research Topic presents three articles. The first article, conducted by Chen et al., introduces a rapid, simple, and equipment-free method for detecting a parasite known as Anisakid. This parasite, hosted in marine foods, can contaminate consumers through the consumption of raw or undercooked foods. The symptoms include gastric, intestinal, ectopic, and allergic responses. Therefore, we expected that this study would contribute to reduction of contamination cases since it presents an affordable methodology for implementation.

In another study featured in this Research Topic, conducted by Zhang et al., researchers employed a rapid detection method using real-time loop-mediated isothermal amplification to identify *Pseudomonas lurida*. This microorganism poses a threat to the quality of milk and dairy products because it produces thermostable alkaline proteases, leading to milk deterioration. Detecting this organism swiftly is crucial for maintaining the high quality of dairy industries products.

The manuscript conducted by Tan et al., focuses on the epidemiological characteristic of *Brucella* in Guizhou province, China. This study was specifically categorized in category four (pathogenic cases in human and animal). Despite mandatory reporting of *Brucella* contamination in China since 1955, the cases have become increasingly frequent and severe in the population, as highlighted by the authors. Additionally, the study revealed a new sequence type (ST39) not previously identified in China. Furthermore, the researchers identified two other MLST genotypes of *Brucella* in the analyzed samples.

Finally, the study performed by Grouteau et al. analyzed strains of *Campylobacter fetus* belonging to a case of food outbreak in elderly people who were in a rehabilitation center. This study demonstrates that failures in the production process and monitoring the quality of food products can lead to serious consequences for consumers, especially risk groups such as children and the elderly, due to the ability to cause foodborne issues in pathogenic strains. In general, this study summarizes that errors during the “farm gate to food plate” can have especially serious consequences in people with compromised immunity.

In conclusion, we emphasize that this Research Topic aimed to explore various aspects without limiting itself to a specific topic. Its objective was demonstrated that the field of food microbiology plays a crucial role in different stages of the food production process. Moreover, global scientific efforts can effectively address regional contamination issues, given the interconnected nature of the food production chain across continents and countries.
